# Supplementary material for: The interactome of intact mitochondria by cross-linking mass spectrometry provides evidence for coexisting respiratory supercomplexes
Source: Mol Cell Proteomics. 2017 Dec 8;17(2):216–32. doi: 10.1074/mcp.RA117.000470 (PMC5795388; doi:10.1074/mcp.RA117.000470)
Supplement: Supplemental Data [file supp_RA117.000470_133922_0_supp_23978_fzffwf.zip › spectra_annotation/mito_DR_spectra_annotation/200-1-5-1-13-1.pdf]

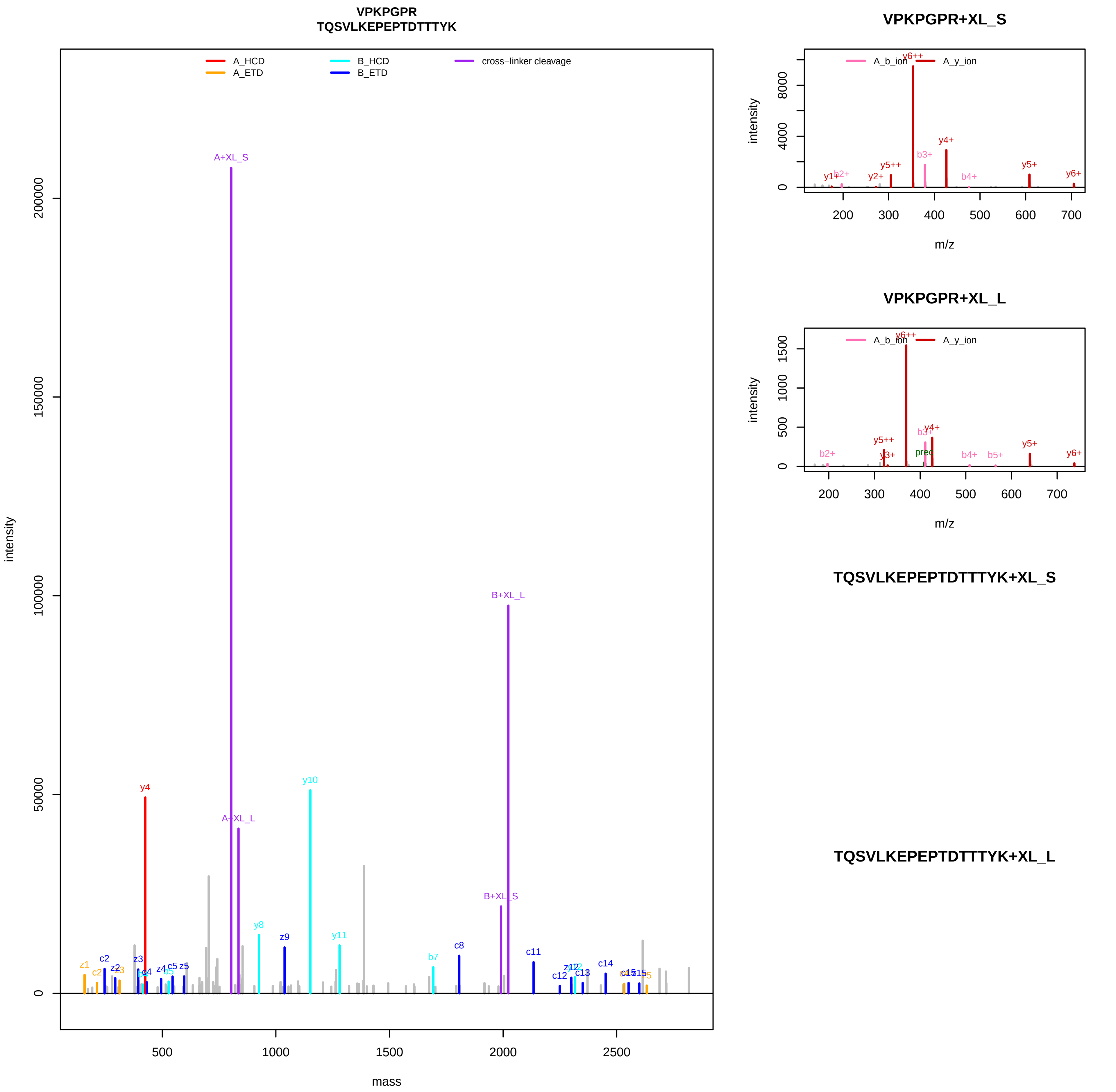
VPKPGPR
TQSVLKEPEPTDTTTYK
A_HCD
A_ETD
B_HCD
B_ETD
cross−linker cleavage
A+XL_S
B+XL_L
A+XL_L
B+XL_S
y4
y10
y8
z9
y11
b7
c8
c11
c12
z12
c13
c14
c15
z15
c2
z1
c2
z2
z3
z3
c4
z4
c5
z5
b5
intensity
mass
VPKPGPR+XL_S
VPKPGPR+XL_L
A_b_ion
A_y_ion
y6++
y4+
b3+
y5++
y5+
y6+
y1+
b2+
y2+
b4+
y3+
b5+
prec
m/z
TQSVLKEPEPTDTTTYK+XL_S
TQSVLKEPEPTDTTTYK+XL_L
